# From curiosity to continuance: explaining nursing students’ continuance learning intention in virtual simulation using an integrated ECM-TAM

**DOI:** 10.1186/s12912-026-04466-6

**Published:** 2026-02-27

**Authors:** Yuan Jiang, Shijie Wu, Minghao Kong

**Affiliations:** 1 Shanghai Tenth People’s Hospital, Tongji University School of Medicine, Shanghai, China; 2 Tongji University School of Medicine, Shanghai, China

**Keywords:** Virtual simulation, Continuance learning intention, Clinical curiosity, Nursing students, PLS-SEM

## Abstract

**Background:**

Virtual simulation is integral to modern nursing education, yet the “acceptance-discontinuance anomaly”—where students cease usage after initial adoption, resulting in fragmented skill acquisition—remains a challenge. Understanding the determinants of Continuance Learning Intention (CLI) is crucial for sustainable competency development. This study aims to investigate the factors influencing nursing students’ CLI toward virtual simulation by integrating the Expectation-Confirmation Model (ECM), Technology Acceptance Model (TAM), and the construct of Clinical Curiosity.

**Methods:**

A descriptive cross-sectional study was conducted at a medical university in Shanghai, Eastern China. A total of 281 nursing students with experience in virtual simulation were recruited to complete an online survey. Data were analyzed using Partial Least Squares Structural Equation Modeling (PLS-SEM) to test the hypothesized relationships among confirmation, perceived clinical usefulness, satisfaction, attitude, clinical curiosity, and CLI.

**Results:**

The integrated model explained 43.8% of the variance in CLI. The model demonstrated good fit with a Standardized Root Mean Square Residual (SRMR) of 0.053. Confirmation significantly predicted Perceived Clinical Usefulness (PCU) and Satisfaction (*p* < 0.001). Satisfaction (β = 0.341, *p* < 0.001) and Attitude (β = 0.402, *p* < 0.001) were direct predictors of CLI. Notably, Clinical Curiosity did not directly predict intention (β = 0.045, *p* = 0.323). Instead, mediation analysis revealed that Attitude fully mediated the relationship between Clinical Curiosity and CLI (β = 0.084, *p* < 0.001).

**Conclusions:**

Clinical relevance and user satisfaction are foundational for retention. Uniquely, intrinsic clinical curiosity alone does not guarantee continued learning in this high-stakes educational context; it must be catalyzed into a positive evaluative attitude to drive sustainable usage. Instructional designs should prioritize bridging curiosity-triggering content with attitude-reinforcing debriefings to foster long-term engagement.

**Supplementary Information:**

The online version contains supplementary material available at 10.1186/s12912-026-04466-6.

## Introduction

The integration of information technology into nursing education has revolutionized traditional pedagogical methods [1–3]. Especially in the post-pandemic era, the reliance on digital simulations to supplement clinical hours has intensified [4, 5]. Virtual simulation systems, which offer risk-free environments for clinical skill acquisition and decision-making practice, have been widely adopted [6, 7]. However, despite substantial investment in these technologies, educational institutions often face the “acceptance-discontinuance anomaly”—a phenomenon where users discontinue using an e-learning system after initially accepting it due to diminishing engagement or perceived utility [8, 9]. For nursing education, this discontinuity is particularly detrimental as it leads to fragmented skill acquisition and undermines the long-term consolidation of clinical skills [10, 11]. Therefore, shifting the research focus from initial adoption to continuance learning intention is imperative.

Existing literature has extensively employed the Expectation-Confirmation Model (ECM) and the Technology Acceptance Model (TAM) to explain information system continuance [8, 12, 13]. Lee [9] successfully synthesized ECM, TAM, and Theory of Planned Behavior to predict e-learning continuance, identifying satisfaction and Perceived Clinical Usefulness (PCU) as key determinants. However, nursing education is distinct from general e-learning; it is driven not only by extrinsic utility but also by intrinsic motivation specific to the profession, such as the desire to understand disease mechanisms [14]. This intrinsic drive aligns with Self-Determination Theory, which posits that intrinsic motivation is a robust predictor of educational persistence [15]. Dai et al. [16] highlighted “curiosity” as a vital intrinsic motive in the MOOC setting, arguing that the drive to know is essential for learning persistence.

Despite these advances, few studies have integrated Clinical Curiosity into the ECM-TAM framework within the specific context of nursing simulation. Furthermore, the mechanism by which curiosity translates into continuance intention remains debated. While Dai et al. [16] proposed a direct link, the high-stakes nature of medical education suggests a different mechanism. We propose that the unique cognitive demands of clinical learning require curiosity to be cognitively processed and mediated by evaluative attitudes before driving sustained behavior. This study fills this gap by proposing a modified model to explore how confirmation, Perceived Clinical Usefulness (PCU), satisfaction, attitude, and clinical curiosity jointly shape nursing students’ continuance learning intention.

## Literature review and hypotheses

### The integrated theoretical framework

**Fig. 1:**
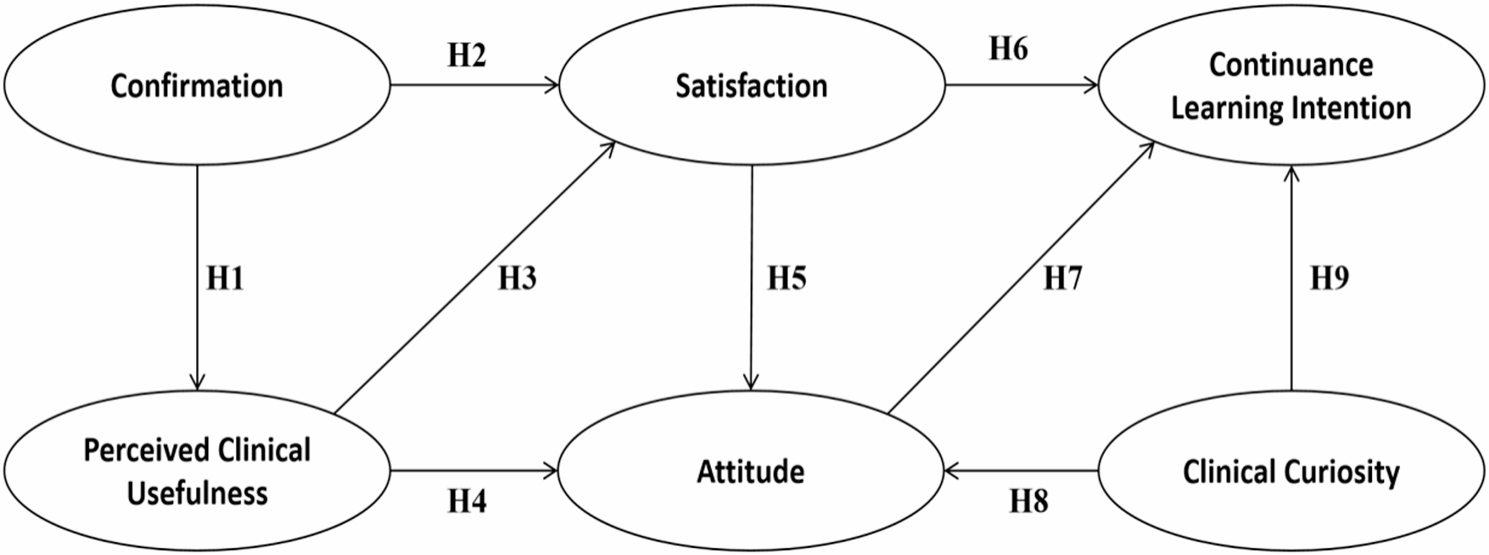
The proposed theoretical model and hypothesized relationships

This study integrates the ECM and TAM, extended with the construct of Clinical Curiosity. The ECM posits that continuance intention is determined by satisfaction, which is influenced by confirmation and ex-post expectations (Perceived Clinical Usefulness). TAM complements this by emphasizing the role of Attitude [12]. Recent studies in healthcare education have increasingly adopted hybrid models to explain complex user behaviors [17–19]. As shown in Fig. 1, the proposed integrated ECM–TAM framework illustrates the hypothesized relationships among confirmation, Perceived Clinical Usefulness (PCU), satisfaction, attitude, clinical curiosity, and continuance learning intention.

### Confirmation, perceived clinical usefulness, and satisfaction

In the ECM, confirmation represents the congruence between expectation and actual performance. Lee [9] demonstrated that confirmation significantly influences both satisfaction and Perceived Clinical Usefulness (PCU). In nursing simulation, if the system accurately reflects clinical realities (Confirmation), students are likely to perceive it as valuable for their practice (Perceived Clinical Usefulness, PCU) and feel satisfied. This relationship has been supported in various e-learning contexts [20].

#### H1

Confirmation is positively related to Perceived Clinical Usefulness.

#### H2

Confirmation is positively related to Satisfaction.

#### H3

Perceived Clinical Usefulness is positively related to Satisfaction.

### The role of attitude and continuance intention

While Lee [9] viewed satisfaction as the primary predictor of intention, Dai et al. [16] argued for distinguishing between Satisfaction (retrospective affect) and Attitude (future-oriented evaluative stance). They posited that Attitude is a more immediate antecedent of intention and is shaped by Satisfaction and Perceived Clinical Usefulness (PCU) [12, 16]. Empirical evidence suggests that positive attitudes towards simulation technology are strong predictors of continued usage among nursing students [21].

#### H4

Perceived Clinical Usefulness is positively related to Attitude.

#### H5

Satisfaction is positively related to Attitude.

#### H6

Satisfaction is positively related to Continuance Learning Intention.

#### H7

Attitude is positively related to Continuance Learning Intention.

### Clinical curiosity as an intrinsic driver

Curiosity, defined as the desire to seek knowledge, is a critical intrinsic motivator in education [22]. Dai et al. [16] incorporated curiosity into the ECM, suggesting it predicts continuance intention. In nursing, “Clinical Curiosity” specifically refers to the epistemic drive to explore clinical mechanisms [23]. Unlike general epistemic curiosity, which is a broad desire for new information, Clinical Curiosity is defined as a context-specific motivational state directed toward resolving medical uncertainties and understanding complex pathological mechanisms [24]. It represents a professional epistemic drive essential for diagnostic reasoning, distinguishing it from mere novelty-seeking. We posit that this curiosity fosters a positive attitude toward the learning system and drives usage.

#### H8

Clinical Curiosity is positively related to Attitude.

#### H9

Clinical Curiosity is positively related to Continuance Learning Intention.

## Method

### Study design

A descriptive cross-sectional study design was employed to investigate the determinants of continuance learning intention among nursing students. The reporting of this study follows the Strengthening the Reporting of Observational Studies in Epidemiology (STROBE) guidelines. The study was conducted at a medical university in Shanghai, Eastern China.

### Participants

The study was conducted in accordance with the Declaration of Helsinki and was approved by the Ethics Committee of the School of Nursing, Tongji University (approval date: April 1, 2025). Written informed consent was obtained from all participants. A survey was conducted among nursing students at a medical university in Shanghai, Eastern China. The inclusion criteria required participants to have completed at least one module of a virtual simulation course. A total of 281 valid responses were obtained after screening for incomplete data. The minimum sample size was calculated using G*Power 3.1 for a multiple regression model (fixed model, R² deviation from zero). The calculation was conservatively based on five predictors, reflecting the maximum number of structural paths pointing at an endogenous construct in the proposed model, to avoid underestimation of the required sample size. Assuming a medium effect size (f² = 0.15), an alpha error probability of 0.05, and a statistical power (1 − β) = 0.95, the required minimum sample size was 138. Therefore, the final sample size of 281 exceeds this threshold and ensures adequate statistical power [25].

Table 1 presents the demographic characteristics of the participants. The sample consisted predominantly of female students, which is consistent with the gender distribution commonly observed in nursing education programs. Participants were drawn from multiple academic years, with the largest proportion being second- and third-year students, reflecting the stage at which virtual simulation courses are most frequently integrated into the curriculum.

**Table 1:** Demographic characteristics of participants (*N* = 281)

Characteristic	Category	Frequency (*N*)	Percentage (%)
Gender	Female	236	84.0%
	Male	45	16.0%
Grade	First year	58	20.6%
	Second year	92	32.7%
	Third year	87	31.0%
	Fourth year	44	15.7%

### Data collection

Data were collected via an online survey platform. The survey link was distributed to eligible students through class social media groups. Informed consent was obtained electronically from all participants before they could access the questionnaire. The survey was anonymous, and participants were informed of their right to withdraw at any time.

### Instruments

All items were measured using a 7-point Likert scale (1 = strongly disagree, 7 = strongly agree). The measurement instrument was adapted from validated scales in previous studies to fit the context of nursing virtual simulation. To ensure cross-cultural applicability, a translation and back-translation procedure was followed [26]. The original English scales were translated into Chinese by two bilingual nursing researchers and then back-translated by a third independent translator to ensure semantic equivalence. Furthermore, a panel of five experts in nursing education and medical informatics reviewed the content validity of the Chinese version to ensure the items accurately captured the constructs within the local context. The specific items and their sources are detailed in Table 2.

**Confirmation (CON), Satisfaction (SAT), Continuance Learning Intention (CLI)**: Adapted from Bhattacherjee [8] and Lee [9].

**Perceived Clinical Usefulness (PCU)**: Adapted from Davis [12] and contextualized to emphasize clinical skill improvement and problem-solving efficiency.

**Attitude (ATT)**: Adapted from Davis [12], focusing on the evaluation of using the system as a “wise choice” or “good idea”.

**Clinical Curiosity (CC)**: Adapted from Litman and Spielberger [22] and Dai et al. [16], focusing on epistemic curiosity regarding clinical diseases and mechanisms.

Prior to formal data collection, the questionnaire was reviewed for clarity and readability by a small group of nursing students, and minor wording refinements were made.

### Data analysis

Partial Least Squares Structural Equation Modeling (PLS-SEM) was employed using SmartPLS 4 software [27]. PLS-SEM is suitable for testing complex models with non-normal data and is widely used in exploratory theory building [28].

## Results

### Measurement model assessment

The measurement model was evaluated for reliability and validity. First, indicator reliability was examined; as shown in Table 2, all item factor loadings ranged from 0.853 to 0.906, exceeding the recommended threshold of 0.708 [28]. Second, internal consistency was established. The Cronbach’s Alpha values ranged from 0.852 to 0.901, and Composite Reliability (CR) values ranged from 0.910 to 0.931, with all constructs surpassing the 0.70 benchmark. Finally, convergent validity was confirmed, as the Average Variance Extracted (AVE) for all constructs ranged from 0.765 to 0.811, well above the 0.50 requirement [29].

Furthermore, the overall model fit was assessed using the Standardized Root Mean Square Residual (SRMR). The SRMR value of the estimated model was 0.053, which is below the recommended threshold of 0.08, indicating a good model fit [30]. The Normed Fit Index (NFI) was 0.883, further supporting the model’s adequacy.

**Table 2:** Construct reliability, validity, and measurement items

Construct	Code	Items Statement	VIF	Factor Loading	Cronbach’s Alpha	CR	AVE
Attitude	ATT1	Using the virtual simulation system for learning is a good idea.	2.009	0.873	0.852	0.91	0.771
	ATT2	Using the virtual simulation system for learning is a wise choice.	2.116	0.874			
	ATT3	I like the idea of using the virtual simulation system for learning.	2.16	0.887			
Clinical Curiosity	CC1	The cases in the virtual simulation system make me want to learn more about related clinical diseases.	2.528	0.875	0.898	0.929	0.765
	CC2	Using the virtual simulation system stimulates my curiosity to further explore clinical nursing knowledge.	2.442	0.881			
	CC3	When I encounter difficulties in the system, I actively want to understand the underlying clinical mechanisms.	2.301	0.853			
	CC4	Using the virtual simulation system makes me intrigued to solve the clinical puzzles presented.	2.784	0.889			
Continuance Learning Intention	CLI1	I intend to continue using the virtual simulation system for learning in the future.	2.271	0.889	0.866	0.918	0.789
	CLI2	I will frequently use the virtual simulation system to reinforce or review my clinical skills if possible.	2.538	0.906			
	CLI3	Even without course or instructor requirements, I would continue using the virtual simulation system for learning.	2.07	0.869			
Confirmation	CON1	My experience with using the virtual simulation system was better than what I expected.	2.439	0.901	0.883	0.928	0.811
	CON2	The functions and learning support provided by the virtual simulation system were better than I expected.	2.539	0.902			
	CON3	The virtual simulation system met my learning requirements better than I expected.	2.488	0.899			
Perceived Clinical Usefulness	PCU1	Using the virtual simulation system helps me improve my clinical nursing skills.	2.164	0.887	0.853	0.911	0.773
	PCU2	Using the virtual simulation system increases my efficiency in solving clinical problems.	2.129	0.882			
	PCU3	I find the virtual simulation system useful for my future clinical practice or internship.	2.02	0.868			
Satisfaction	SAT1	I am satisfied with my overall experience of using the virtual simulation system.	2.411	0.868	0.901	0.931	0.771
	SAT2	I feel pleased with my experience of using the virtual simulation system.	2.757	0.892			
	SAT3	Overall, my experience of using the virtual simulation system has been satisfactory.	2.615	0.883			
	SAT4	I feel contented with my experience of using the virtual simulation system.	2.453	0.869			

Discriminant validity was assessed using the Heterotrait-Monotrait ratio (HTMT). As presented in Table 3, all HTMT values were below the conservative threshold of 0.85 [31], confirming that the constructs are empirically distinct. Furthermore, Harman’s single-factor test revealed that the first factor explained 38.55% of the total variance, which is below the 50% threshold [32], suggesting that common method bias was not a critical issue in this study.

**Table 3:** Discriminant validity (HTMT)

ATT	ATT	CC	CLI	CON	PCU	SAT
CC	0.381					
CLI	0.685	0.299				
CON	0.388	0.32	0.403			
PCU	0.507	0.207	0.462	0.465		
SAT	0.573	0.272	0.628	0.544	0.537	

### Structural model assessment

The structural model was assessed for explanatory power and path significance. The model explained 43.8% of the variance in Continuance Learning Intention (R^2^ = 0.438), 34.3% in Attitude, and 32.6% in Satisfaction, indicating moderate to substantial predictive power [28].

**Fig. 2:**
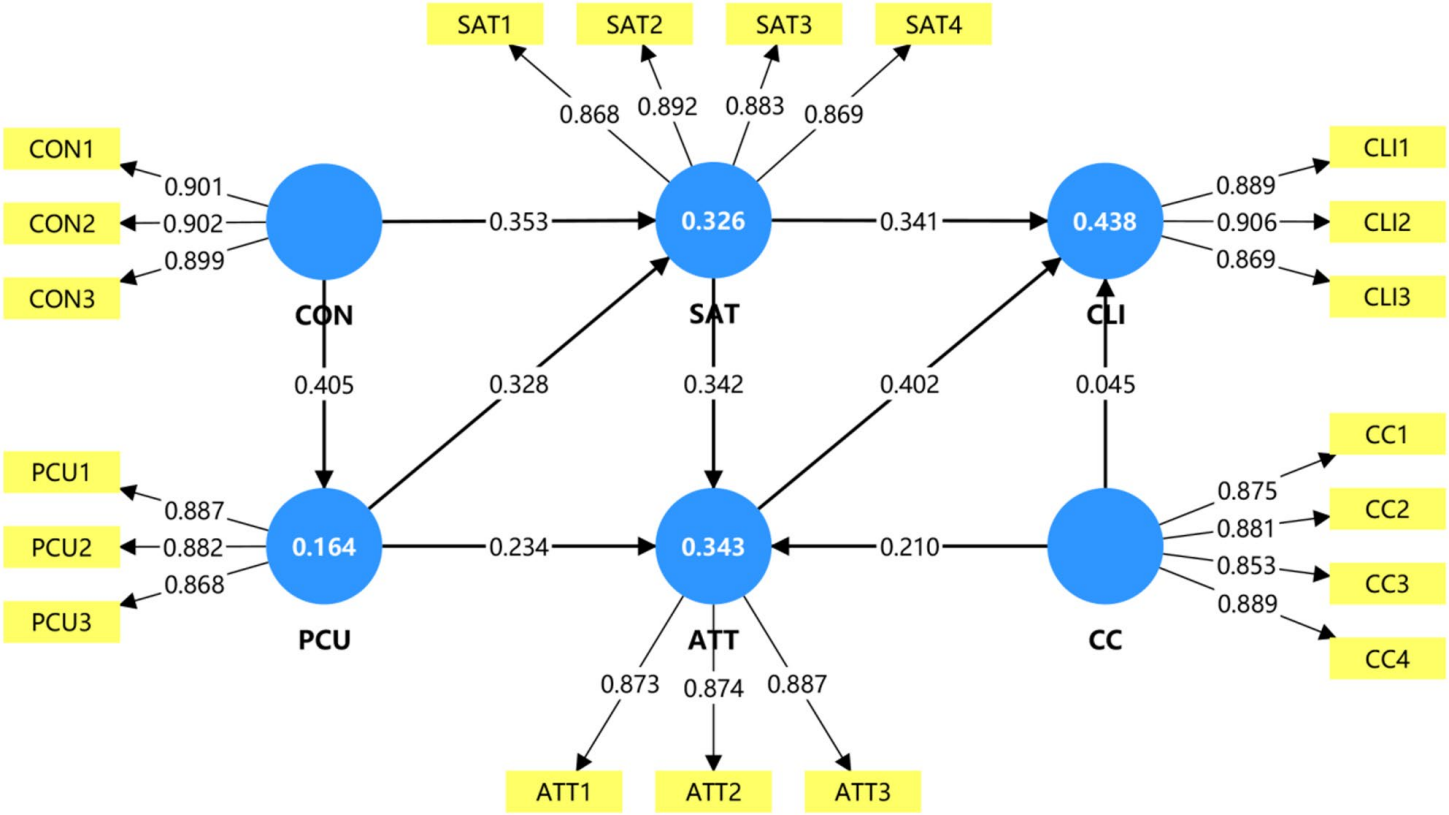
Structural model results with path coefficients

Hypothesis testing was conducted using a bootstrapping procedure with 5,000 subsamples. As shown in Fig. 2; Tables 4 and 8 out of 9 hypotheses were supported.

Confirmation had strong positive effects on PCU (β = 0.405, *p* < 0.001) and Satisfaction (β = 0.353, *p* < 0.001), supporting H1 and H2.

PCU significantly influenced Satisfaction (β = 0.327, *p* < 0.001) and Attitude (β = 0.233, *p* < 0.001), supporting H3 and H4.

Satisfaction significantly predicted Attitude (β = 0.343, *p* < 0.001) and CLI (β = 0.341, *p* < 0.001), supporting H5 and H6.

Attitude was a significant predictor of CLI (β = 0.402, *p* < 0.001), supporting H7.

Clinical Curiosity (CC) significantly predicted Attitude (β = 0.212, *p* < 0.001), supporting H8. However, CC did not have a significant direct effect on CLI (β = 0.045, *p* = 0.323). Therefore, H9 was not supported.

**Table 4:** Structural model results and hypothesis

Hypothesis	Relationship	Beta (β)	mean	SD	T statistics	*P* values	f²	Decision
H1	CON -> PCU	0.405	0.407	0.045	9.057	<0.001	0.196	Supported
H2	CON -> SAT	0.353	0.355	0.055	6.398	<0.001	0.154	Supported
H3	PCU -> SAT	0.328	0.327	0.053	6.178	<0.001	0.134	Supported
H4	PCU -> ATT	0.234	0.233	0.054	4.327	<0.001	0.064	Supported
H5	SAT -> ATT	0.342	0.343	0.051	6.706	<0.001	0.133	Supported
H6	SAT -> CLI	0.341	0.34	0.052	6.521	<0.001	0.153	Supported
H7	ATT -> CLI	0.402	0.403	0.054	7.49	<0.001	0.201	Supported
H8	CC -> ATT	0.21	0.212	0.049	4.284	<0.001	0.063	Supported
H9	CC -> CLI	0.045	0.046	0.046	0.988	0.323	0.003	Not Supported

### Mediation analysis

Given the non-significant direct path from Clinical Curiosity to Intention, a specific indirect effects analysis was performed [33]. The results revealed that the indirect path CC → ATT → CLI was statistically significant (β = 0.084). This finding indicates that Attitude fully mediates the relationship between Clinical Curiosity and Continuance Learning Intention. Additionally, the path SAT → ATT → CLI (β = 0.137) and PCU → SAT → CLI (β = 0.112) were also significant, highlighting the multiple mediating roles of Attitude and Satisfaction. The results are presented in Table 5.

**Table 5:** Specific indirect effects analysis results

Relationship	Beta (β)	mean	SD	T statistics	*P* values
CC -> ATT-> CLI	0.084	0.086	0.024	3.492	0.000
SAT -> ATT-> CLI	0.137	0.138	0.026	5.188	0.000
PCU -> SAT-> CLI	0.112	0.112	0.027	4.218	0.000
PCU -> ATT-> CLI	0.094	0.095	0.027	3.428	0.001
CON -> PCU-> SAT	0.133	0.133	0.027	5.008	0.000
CON -> SAT-> CLI	0.12	0.121	0.026	4.583	0.000
CON -> SAT-> ATT	0.12	0.122	0.028	4.358	0.000
CON -> PCU-> ATT	0.095	0.095	0.024	3.929	0.000
PCU -> SAT-> ATT	0.112	0.112	0.024	4.581	0.000
CON -> SAT -> ATT -> CLI	0.048	0.049	0.013	3.681	0.000
CON -> PCU -> ATT -> CLI	0.038	0.038	0.012	3.208	0.001
PCU -> SAT -> ATT -> CLI	0.045	0.045	0.011	4.097	0.000
CON -> PCU -> SAT -> CLI	0.045	0.046	0.012	3.699	0.000
CON -> PCU -> SAT -> ATT	0.045	0.046	0.011	3.98	0.000
CON -> PCU -> SAT -> ATT -> CLI	0.018	0.018	0.005	3.638	0.000

### Predictive power assessment

To examine the predictive relevance of the proposed model, we applied the PLS-Predict algorithm (Shmueli et al., 2016; Shmueli et al., 2019). Unlike the traditional R^2^ which indicates in-sample explanatory power, PLS-Predict assesses the model’s out-of-sample predictive power using a fold-based procedure.

As shown in Table 6, the Q^2^ values for all endogenous constructs (ATT, CLI, PCU, and SAT) are positive (> 0), ranging from 0.142 to 0.228. According to Shmueli et al. (2019), positive Q^2^ values indicate that the model’s prediction error is smaller than that of a naive mean-based benchmark. Therefore, the model demonstrates sufficient predictive relevance for the key constructs, particularly for Satisfaction (Q^2^ = 0.228) and Continuance Learning Intention (Q^2^ = 0.142).

**Table 6:** Predictive power assessment

	Q²predict	RMSE	MAE
ATT	0.166	0.921	0.732
CLI	0.142	0.933	0.743
PCU	0.156	0.926	0.731
SAT	0.228	0.885	0.714

## Discussion

### General discussion

This study successfully validated an integrated ECM-TAM model tailored to nursing education. The results largely echo the findings of Lee [9], confirming that Satisfaction and Attitude are the primary proximal determinants of continuance intention. The strong influence of Confirmation on both Perceived Clinical Usefulness (PCU) and Satisfaction underscores that the “first impression”—specifically whether the system meets clinical expectations—is the cornerstone of long-term retention. This aligns with recent research emphasizing the importance of user satisfaction in sustaining mobile health application usage [18]. Similar dependencies between system quality expectations and user satisfaction have been observed in recent augmented reality (AR) training studies [34, 35].

### The mediation of attitude on curiosity

A distinct contribution of this study is the clarification of how Clinical Curiosity influences retention. Contrary to Dai et al. [16], who found a direct link between curiosity and intention in MOOCs, our results in the nursing simulation context support a full mediation model. This full mediation suggests a critical boundary condition in professional education. Unlike general interest-driven learning (e.g., MOOCs) where curiosity directly fuels persistence, nursing education is highly pragmatic and goal-oriented. Clinical curiosity (“I want to know the mechanism”) acts as an initial trigger, but it must be cognitively processed into a positive evaluative attitude (“Using this system is a wise professional investment”) to drive sustainable usage. Without this attitudinal transformation, raw curiosity may fade quickly under the pressure of academic workload. This finding refines the application of intrinsic motivation theories in medical education [14, 23], suggesting that educators must not only trigger curiosity but also guide students to form positive evaluative stances toward the learning tools. This contrasts with informal learning settings where curiosity might directly drive exploration [36].

### The role of clinical usefulness

The significant impact of Perceived Clinical Usefulness on both Satisfaction and Attitude validates our contextualization of the TAM construct. For nursing students, a system is only “useful” if it enhances clinical decision-making and efficiency. This pragmatic orientation aligns with the vocational nature of nursing education and supports previous findings on the utility-centric adoption of technology by healthcare professionals [13, 21].

## Implications

### Theoretical implications

First, this study extends the ECM and TAM frameworks by validating the construct of Perceived Clinical Usefulness, demonstrating that context-specific utility is crucial for model explanatory power. Second, it elucidates the mechanism of epistemic curiosity. By establishing the full mediation of Attitude, this research provides a more nuanced understanding of how intrinsic traits translate into behavioral intention in high-stakes educational environments.

### Practical implications

For Educators: To boost retention, instructional design should move beyond mere content delivery to triggering Clinical Curiosity (e.g., through complex, unsolved case studies). However, educators must also facilitate debriefing sessions to help students internalize the value of the simulation, thereby fostering a positive attitude.

For System Developers: The strong dependency of Satisfaction on Confirmation suggests that marketing and instructional claims must be realistic. Over-promising and under-delivering will severely damage satisfaction and, consequently, retention.

### Limitations and future study

This study has several limitations. First, the cross-sectional design precludes causal inferences; longitudinal studies are needed to examine how continuance intention translates into actual usage behavior over time. Second, the data were based on self-reported measures, which may introduce common method bias; future research could incorporate objective system log data or multi-source assessments. Third, the study was conducted in a single university setting, which may limit generalizability. Although institutional homogeneity may reduce contextual variability in theory-testing studies employing PLS-SEM, the findings should be interpreted with caution. The curriculum at our institution follows the National Standards for Nursing Education in China; however, replication across multiple institutions and diverse cultural contexts is warranted to further examine the robustness and generalizability of the proposed model.

## Supplementary Information

Below is the link to the electronic supplementary material.

Supplementary Material 1

## Data Availability

The datasets generated and/or analyzed during the current study are not publicly available due to privacy restrictions but are available from the corresponding author on reasonable request.

## Abbreviations

ECM: Expectation-Confirmation Model
TAM: Technology Acceptance Model
CLI: Continuance Learning Intention
PCU: Perceived Clinical Usefulness
CON: Confirmation
SAT: Satisfaction
ATT: Attitude
CC: Clinical Curiosity
