# Supplementary material for: From curiosity to continuance: explaining nursing students’ continuance learning intention in virtual simulation using an integrated ECM-TAM
Source: BMC Nurs. 2026 Feb 27;25:305. doi: 10.1186/s12912-026-04466-6 (PMC13040937; doi:10.1186/s12912-026-04466-6)
Supplement: Supplementary file 1 — Supplementary Material 1 [file 12912_2026_4466_MOESM1_ESM.docx]

**Supplementary File 1.** Survey Instrument

Note: All items were measured using a 7-point Likert scale ranging from 1 “Strongly Disagree” to 7 “Strongly Agree”. The instrument was adapted from validated scales in previous studies and contextualized for the nursing virtual simulation setting.

Table S1. Measurement Items and Sources

| **Variables** | **Code** | **Item Statement** | **Source** |
| --- | --- | --- | --- |
| Confirmation | CON1 | My experience with using the virtual simulation system was better than what I expected. | Adapted from Bhattacherjee, 2001; Lee, 2010 |
|  | CON2 | The functions and learning support provided by the virtual simulation system were better than I expected. |  |
|  | CON3 | The virtual simulation system met my learning requirements better than I expected. |  |
| Perceived Clinical Usefulness | PCU1 | Using the virtual simulation system helps me improve my clinical nursing skills. | Adapted from Davis, 1989 |
|  | PCU2 | Using the virtual simulation system increases my efficiency in solving clinical problems. |  |
|  | PCU3 | I find the virtual simulation system useful for my future clinical practice or internship. |  |
| Satisfaction | SAT1 | I am satisfied with my overall experience of using the virtual simulation system. | Adapted from Bhattacherjee, 2001; Lee, 2010 |
|  | SAT2 | I feel pleased with my experience of using the virtual simulation system. |  |
|  | SAT3 | Overall, my experience of using the virtual simulation system has been satisfactory. |  |
|  | SAT4 | I feel contented with my experience of using the virtual simulation system. |  |
| Attitude | ATT1 | Using the virtual simulation system for learning is a good idea. | Adapted from Davis, 1989 |
|  | ATT2 | Using the virtual simulation system for learning is a wise choice. |  |
|  | ATT3 | I like the idea of using the virtual simulation system for learning. |  |
| Clinical Curiosity | CC1 | The cases in the virtual simulation system make me want to learn more about related clinical diseases. | Adapted from Litman & Spielberger, 2003; Dai et al., 2020 |
|  | CC2 | Using the virtual simulation system stimulates my curiosity to further explore clinical nursing knowledge. |  |
|  | CC3 | When I encounter difficulties in the system, I actively want to understand the underlying clinical mechanisms. |  |
|  | CC4 | Using the virtual simulation system makes me intrigued to solve the clinical puzzles presented. |  |
| Continuance Learning Intention | CLI1 | I intend to continue using the virtual simulation system for learning in the future. | Adapted from Bhattacherjee, 2001; Lee, 2010 |
|  | CLI2 | I will frequently use the virtual simulation system to reinforce or review my clinical skills if possible. |  |
|  | CLI3 | Even without course or instructor requirements, I would continue using the virtual simulation system for learning. |  |
